# Inhibition of p38MAPK and CD137 signaling reduce dengue virus-induced TNF-α secretion and apoptosis

**DOI:** 10.1186/1743-422X-10-105

**Published:** 2013-04-04

**Authors:** Amar Nagila, Janjuree Netsawang, Aroonroong Suttitheptumrong, Atthapan Morchang, Sasiprapa Khunchai, Chatchawan Srisawat, Chunya Puttikhunt, Sansanee Noisakran, Pa-thai Yenchitsomanus, Thawornchai Limjindaporn

**Affiliations:** 1 Division of Molecular Medicine, Department of Research and Development, Faculty of Medicine Siriraj Hospital, Mahidol University, Bangkok, Thailand; 2 Department of Biochemistry, Faculty of Medicine Siriraj Hospital, Mahidol University, Bangkok, Thailand; 3 Department of Anatomy, Faculty of Medicine Siriraj Hospital, Mahidol University, Bangkok, Thailand; 4 Department of Immunology, Faculty of Medicine Siriraj Hospital, Mahidol University, Bangkok, Thailand; 5 Faculty of Medical Technology, Rangsit University, Bangkok, Thailand; 6 Medical Biotechnology Research Unit, National Center for Genetic Engineering and Biotechnology, National Science and Technology Development Agency, Bangkok, Thailand

**Keywords:** Dengue virus, Apoptosis, TNF-α, p38MAPK, CD137

## Abstract

**Background:**

Hepatic injury in dengue virus (DENV) infection is authenticated by hepatomegaly and an upsurge in transaminase levels. DENV replicates in hepatocytes and causes hepatocyte apoptosis both *in vitro* and *in vivo*. Understanding the molecular mechanisms of DENV-induced hepatic injury could facilitate the development of alternate chemotherapeutic agents and improved therapies.

**Findings:**

The p38 mitogen-activated protein kinase (MAPK) participates in both apoptosis-related signaling and pro- inflammatory cytokine production. The role of p38 MAPK in DENV-infected HepG2 cells was examined using RNA interference. The results showed that DENV infection activated p38 MAPK and induced apoptosis. The p38 MAPK activation and TNF-α production were controlled by p38 MAPK and CD137 signaling in DENV-infected HepG2 cells as activated p38 MAPK, TNF-α and apoptosis were significantly decreased in p38 MAPK and CD137 depleted DENV-infected HepG2 cells. Addition of exogenous TNF-α to p38 MAPK depleted DENV-infected HepG2 cells restored DENV-induced apoptosis in HepG2 cells.

**Conclusion:**

DENV induces CD137 signaling to enhance apoptosis by increasing TNF-α production via activation of p38 MAPK.

## Findings

Hepatic dysfunction is a crucial feature of DENV infection. Hepatic biopsy specimens obtained from fatal cases of dengue shock syndrome (DSS) show cellular apoptosis, which may relate to the pathogenesis of DSS [1,2]. DENV replicates in hepatocytes and apoptosis of DENV-infected hepatic cells has been observed both *in vitro* and *in vivo*. Both the cell death receptor and the mitochondrial apoptotic pathways are affected [3-9].

DENV capsid protein (DENV C) activates both extrinsic and intrinsic apoptotic pathways in hepatic cell lines [10-12]. DENV C localizes to both the cytoplasm and nucleus of DENV-infected HepG2 cells. DENV C contains three nuclear localization signals (NLS), ^6^KKAR^9^, ^73^KKSK^76^ and the bipartite signal ^85^RKeigrmlnilnRRRR^100^. Substitution mutations in DENV C (R85A/K86A) result in loss of nuclear localization, Daxx interaction, and apoptosis [11]. Comparison of the apoptotic gene expression profile of DENV C and DENV C (R85A/K86A) transfected HepG2 cells showed a significant increase in expression of CD137, a member of TNF receptor family, in HepG2 cells expressing DENV C. In DENV-infected HepG2 cells, CD137 and CD137 ligand mRNA expression increased 60-fold and 3-fold post DENV infection, respectively. CD137 positive cells increased, but less dramatically, about 3-fold post infection and correlates with the level of apoptosis induced by DENV infection of HepG2 cells, which increased 4-fold post DENV infection [13].

CD137 recruits TNF receptor associated factor 2 (TRAF2) and activates apoptosis signal regulating kinase 1 (ASK1), resulting in activation of cJun N-terminal kinase (JNK) and p38 mitogen-activated protein kinase (MAPK) [14]. p38 MAPK is primarily implicated in apoptosis-related signaling [15]. However, activation of p38 MAPK increases inflammatory cytokine production [16]. Activation of p38 MAPK may contribute to the pathogenesis of DENV-induced apoptosis via both mechanisms.

### Inhibition of DENV-induced apoptosis in HepG2 cells by siRNA against p38 MAPK

**Figure 1:**
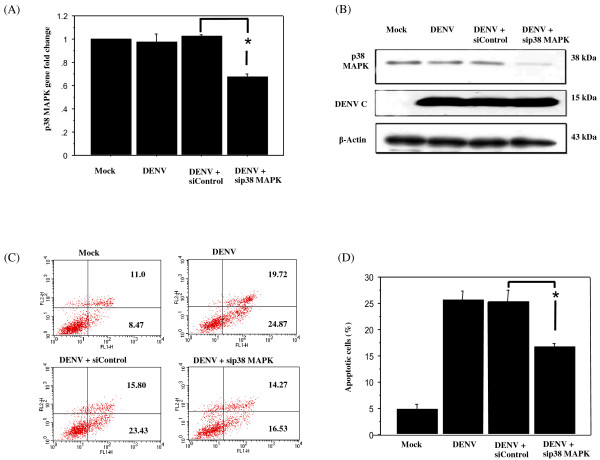
**Decreased apoptosis in p38 MAPK knockdown DENV-infected HepG2 cells.** HepG2 cells were transfected either with siRNA directed against p38 MAPK or with control siRNA. Twenty-four hours post transfection, cells were infected with DENV at MOI 5 for 60 h. Cells were harvested and analyzed for (**A**) mRNA level of p38 MAPK (**B**) protein level of p38 MAPK. (**C**) Apoptosis by flow cytometry. Apoptosis cells have annexin V+/PI- staining. (**D**) Bar graph represents apoptosis experiments. All data were obtained from three independent experiments and reported as the mean ± SEM. Statistical differences between the groups were tested with an unpaired *t*-test using StatView version 5.0 and *P* value less than 0.05 was considered significant.

We first asked whether RNAi knockdown of p38α MAPK, which is a crucial mediator of proinflammatory cytokine production, would inhibit DENV-induced apoptosis in HepG2 cells, an approach that might alleviate DENV-mediated hepatic injury. At 24 h after seeding, HepG2 cells were transfected with 200 pmol of siRNA directed against p38α MAPK, 5^′^CAGACCATATTAACCAGCTTCAGCA3^′^ or with siControl, 5^′^ CACGCCTCTTTGTCTTGTTTCGAAA 3^′^ (Invitrogen), using lipofectamine 2000 (Invitrogen). Twenty-four hours post transfection, the cells were infected with DENV serotype 2 strain 16681 at an MOI of 5 using the methods described previously [13]. The percentage of apoptotic cells was determined by annexin V/FITC and PI double staining (BD Biosciences) and quantitation by flow cytometry. The efficiency of p38α MAPK knockdown by siRNA was examined by real-time RT-PCR using p38α MAPK-specific primers (p38α MAPK-F, 5^′^CGACTTGCTGGAGAAGATGC3^′^, and p38α MAPK-R, 5^′^TCCATCTCTTCTTGGTCAAGG3^′^) and by Western blot analysis using primary antibodies against p38 MAPK and β-actin (Santa Cruz Biotechnology). About 30% decreases in both mRNA and protein expression levels of p38 MAPK were observed (Figure 1A, 1B). In addition, treatment with siRNA against p38 MAPK reduced apoptosis from 23.43% to 16.53% in DENV-infected HepG2 cells (Figure 1C, D) suggesting a role for p38 MAPK in DENV-mediated apoptosis. The result of siRNA directed against p38 MAPK in DENV-infected HepG2 cells is agreeable to our previous study of pharmacological inhibition of p38 MAPK by SB203580 in DENV-infected HepG2 cells [17]. Therefore, genetic inhibition of p38 activity reproduces pharmacological inhibition of the enzyme and confirms the contribution of p38 MAPK to apoptotic events induced by DENV [17]. DENV infected HepG2 cells equally in the presence or absence of SB203580 [17] but DENV production in BHK-21 cells was decreased in the presence of SB203580 [18].

### Inhibition of DENV C-induced apoptosis in stable HepG2 cell expressing DENV C by siRNA against p38 MAPK

**Figure 2:**
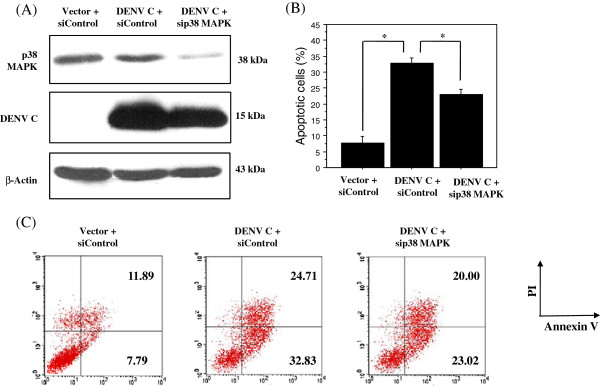
**Decreased apoptosis in p38 MAPK knockdown HepG2 cells expressing DENV C.** HepG2 cells expressing DENV C or control plasmid were transfected either with siRNA directed against p38 MAPK or with control siRNA and then treated with 0.5 μg/ml anti-Fas mAb and 1 ug/ml cycloheximide for 24 h. Cells were collected and analyzed for (**A**) p38 MAPK, DENV C and actin (**B**) Bar graph represents apoptosis experiments. All data were obtained from three independent experiments and reported as the mean ± SEM. Statistical differences between the groups were tested with an unpaired *t*-test using StatView version 5.0 and *P* value less than 0.05 was considered significant. (**C**) Apoptosis by flow cytometry.

We next asked whether DENV C activates p38 MAPK-induced apoptosis by examining the effect of siRNA against p38α MAPK on apoptosis of HepG2 cells stably expressing DENV C. HepG2 cells stably expressing DENV C were constructed in this study using retroviral system (Stratagene). Briefly, 70% confluent of HEK 293T cells was transfected with p-VPack-GP, p-VPack-VSV-G and pFB-Neo-DENV C using lipofectamine 2000 (Invitrogen). The culture supernatant containing infectious viral particles was collected after 24 h post transfection and added to HepG2 cells, which were pre-incubated with 8 μg / ml of polybrene. Twenty-four h after incubation, stable HepG2 cells expressing DENV C were selected with media containing 0.5 mg/ml G418 (Calbiochem). The G418-resistant cells were grown and maintained in DMEM medium containing 0.5 mg/ml G418, and the expression of DENV C was examined by flow cytometry and Western blot analysis using antibody to DENV C [19]. Up to 5 × 10^5^ the stably expressing cells were plated for 24 h prior to transfection. The cells were then transfected with siRNAs as described in the preceding experiment. Knock-down efficiency was assessed by Western blot analysis. The 15 kDa capsid protein was expressed in stable HepG2 cells expressing DENV but not in HepG2 cells expressing control plasmid (Figure 2A). To activate the extrinsic apoptotic pathway, cells were treated with 0.5 μg/ml anti-Fas mAb (Sigma) and 1 ug/ml cycloheximide (Sigma) for 24 h in culture medium. Both adherent and floating cells, as well as culture supernatants, were collected and assessed for apoptosis by annexinV/PI staining (BD Biosciences). As with DENV-infected cells, siRNA p38α MAPK resulted in a substantial reduction in p38 MAPK protein, but had no effect on DENV C expression (Figure 2A). In the presence of anti-Fas and cycloheximide, apoptosis of HepG2 cells expressing DENV C increased from 7.79% to 32.83% compared to that of HepG2 cells expressing control plasmid. HepG2 cells expressing DENV C transfected with p38 MAPK siRNA reduced apoptosis from 32.83% to 23% (Figure 2B, 2C). In our previous study without anti-Fas mAb and cycloheximide treatment, HepG2 cells were transiently transfected with a DENV C or control plasmid and incubated in the presence of DMSO or 10 μM of SB203580 for 24 h. The percentage of apoptotic cells was then determined by annexin V/FITC and PI double staining and quantitation by flow cytometry. Comparable to apoptosis of HepG2 cells expressing control plasmid, apoptosis of HepG2 cells expressing DENV C increased from 10.11% to 24.40%. Apoptosis of HepG2 cells expressing DENV C decreased from 24.40% to 5.69% in medium with SB203580 [17]. Therefore, genetic inhibition of p38 activity reproduces pharmacological inhibition of the enzyme and verifies the contribution of p38 MAPK to apoptotic events induced specifically by DENV C. However, not all DENV-induced cell death is caused by DENV C, other DENV proteins including M, NS3 protease and NS2B-NS3 precursor also induces apoptosis [20,21].

### Inhibition of DENV-induced phosphorylated p38 MAPK, TNF-α production and apoptosis in HepG2 cells by siRNA against CD137

**Figure 3:**
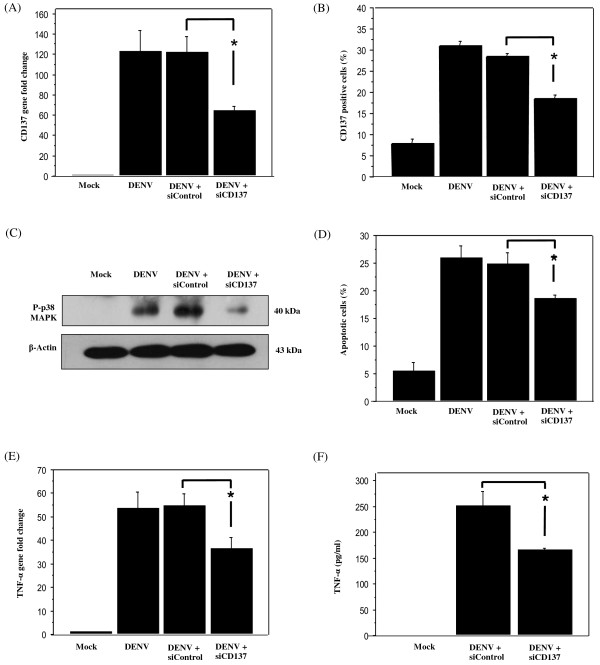
**Reduced DENV-induced phosphorylated p38 MAPK, TNF-αand apoptosis in HepG2 cells by siRNA against CD137.** HepG2 cells were transfected with either CD137 siRNA or control siRNA. Twenty-four hours post transfection, cells were infected with DENV at MOI 5 for 60 h. Cells were collected and analyzed for (**A**) CD137 mRNA (**B**) CD137 positive cells (**C**) phosphorylation of p38 and (**D**) Apoptosis by flow cytometry. The cells were collected and analyzed for TNF-α mRNA (**E**) and the culture supernatant was analyzed for TNF-α protein (**F**). All data were obtained from three independent experiments and reported as the mean ± SEM. Statistical differences between the groups were tested with an unpaired *t*-test using StatView version 5.0 and *P* value less than 0.05 was considered significant.

To further define the molecular mechanisms involved, we asked whether CD137 signaling regulates p38 MAPK activation and apoptosis in DENV-infected HepG2 cells. siRNA knockdown of CD137 was performed as described for p38 MAPK using the CD137-specific oligo 5^′^CACGCTCCGTTTCTCTGTTGTTAAA 3^′^ (Invitrogen). The efficiency of knockdown was examined by real-time RT-PCR using CD137-specific primers CD137-F, 5^′^CCA AAA TGT TCT GCT GAT CG3^′^ and CD137-R, 5^′^ AAG ACT GTG GCG CCC TG3^′^. The number of CD 137 positive cells was measured by flow cytometry using a primary antibody against CD137 (Santa Cruz Biotechnology). Transfection of HepG2 cells with siRNA against CD137 resulted in a nearly 2-fold reduction in CD137 mRNA and CD137-positive cells (Figure 3A, 3B). The effect of CD137 depletion on p38 MAPK activation during DENV infection and apoptosis were measured by Western blot analysis using primary antibody against phosphorylated p38 MAPK (Santa Cruz Biotechnology), and by annexinV/PI staining (BD Biosciences), respectively. Knockdown of CD137 expression reduced the amount of phosphorylated p38 MAPK (Figure 3C) and apoptosis (Figure 3D). These results indicate a role of CD137 signaling in regulation of p38 MAPK activation and apoptosis in DENV-infected HepG2 cells. As DENV induced CD137 expression only 30% of the infected cells (Figure 3B) and expressed at the late stage of infection [13], CD137 and p38 MAPK signaling may support other apoptotic signaling pathways [3-9] to aggravate apoptosis at the late stage of DENV infection. Multiple studies *in vitro* and *in vivo* models implicate TNF-α in DENV-induced tissue damage [8,22]. In addition, TNF-α induced apoptosis via p38 MAPK activation was shown in pseudorabies virus infection [23].

We next asked whether TNF-α mediated DENV-induced apoptosis via CD137 signaling. TNF-α expression in CD137 depleted DENV-infected HepG2 cells was examined by real-time RT-PCR using TNF-α-specific primers TNF-α-F, 5^′^TGCTTGTTCCTCAGCCTCTT3^′^,and TNF-α-R, 5^′^ ATGGGCTACAGGCTTGTCACT3^′^ and by ELISA (R&D Systems). DENV infection of HepG2 cells resulted in a dramatic increase in TNF-α mRNA and protein (Figure 3E, 3F). However, CD137 siRNA treatment reduced of TNF-α production about 30% (Figure 3E, 3F), which correlates well with the comparable reduction in apoptosis of CD137 knockdown cells (Figure 3D).

### Inhibition of p38 MAPK activity by SB203580 decreased TNF-α production in DENV-infected HepG2 cells

**Figure 4:**
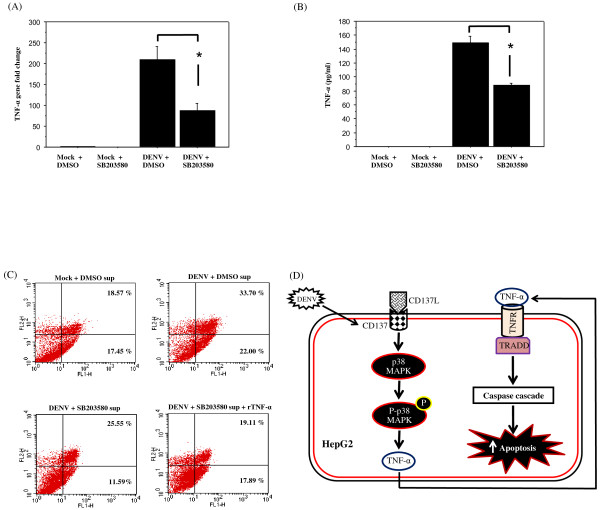
**Reduced DENV-induced TNF-α production and apoptosis in SB203580 treated DENV-infected HepG2 cells and exogenous TNF-α restored the apoptosis.** (**A**) HepG2 cells were infected with DENV serotype 2 at a MOI 5 and incubated 48 h in the presence of either DMSO or 10 μM SB203580. TNF-α mRNA (A) and TNF-α protein (**B**) expression of DENV-infected HepG2 cells was subsequently examined by real-time RT-PCR and by ELISA, respectively. All data were obtained from three independent experiments and reported as the mean ± SEM. Statistical differences between the groups were tested with an unpaired *t*-test using StatView version 5.0 and *P* value less than 0.05 was considered significant. (**C**) Supernatants containing DENV from mock-infected or DENV-infected HepG2 cells in the presence of either DMSO or 10 μM SB203580 was isolated and incubated with HepG2 cells in the presence or absence of recombinant TNF-α. The percentage of apoptotic cells was then determined at 72 h by annexin V/FITC and PI double staining and quantitation by flow cytometry. Apoptosis cells have annexin V+/PI- staining. (**D**) Model representing the augmentation of DENV-induced apoptosis mediated by TNF-α via the activation of p38 MAPK and CD137 signaling.

As p38 MAPK is one of the downstream targets of CD137 signaling, it is important to assess whether inhibition of p38 MAPK activity in DENV-infected HepG2 cells would reduce TNF-α expression. HepG2 cells were infected with DENV serotype 2 at a MOI 5 and incubated 48 h in the presence of either DMSO or 10 μM SB203580 (Santa Cruz Biotechnology). TNF-α mRNA expression of DENV-infected HepG2 cells and TNF-α protein expression in supernatant of DENV-infected HepG2 cells were subsequently examined by real-time RT-PCR and by ELISA, respectively. As expected, treatment with 10 μM SB203580 significantly reduced TNF-α expression in DENV-infected HepG2 cells (Figure 4A, 4B) indicating that pharmacological inhibition of p38 MAPK reduced TNF-α secretion in DENV-infected HepG2 cells. Whether inhibition of both p38 and CD137 signalings has the additive effect on TNF-α production merits further investigation.

### The role of TNF-α expression in apoptosis of DENV-infected HepG2 cells

It would be important to evaluate the effect of TNF-α expression in apoptosis of DENV-infected HepG2 cells. Supernatants containing DENV from mock-infected or DENV-infected HepG2 cells in the presence of either DMSO or 10 μM SB203580 was isolated and further incubated with HepG2 cells in the presence or absence of recombinant TNF-α (250 ng/ml)(Sigma). The percentage of apoptotic cells was then determined at 72 h by annexin V/FITC and PI double staining and quantitation by flow cytometry. The result in Figure 4C showed that apoptosis of DENV-infected HepG2 cells decreased from 22% to 11.59% when HepG2 cells were incubated with supernatant containing virus from SB203580 treated DENV-infected HepG2 cells. Addition of recombinant TNF-α to supernatant from SB203580 treated DENV-infected HepG2 cells increased apoptosis of DENV-infected HepG2 cells from 11.59% to 17.89% (Figure 4C) suggesting that TNF-α production affects DENV-induced apoptosis of HepG2 cells. Our result supports the previous studies for the role of TNF-α expression in DENV-induced apoptosis in other cell lines. TNF-α produced from macrophage was shown to enhance DENV-induced endothelial cell death [24] and inhibition of peripheral blood mononuclear cell (PBMC) apoptosis by etanercept, which is the antibody to TNF-α, was also reported [25]. In summary, we propose in Figure 4D that DENV induces phosphorylated p38 MAPK to stimulate apoptosis and inhibition of p38 MAPK and CD137 pathway reduce DENV-induced TNF-α secretion and apoptosis of HepG2 cells.

## Competing interests

The authors declare that they have no competing interest.

## Authors’ contributions

TL, PY and SN conceived of the study. AN, JN, AM and CS carried out experiments in RNA interference and apoptosis. AS and SK carried out cytokine assay. CP participated in antibody production. All authors have read and approved the final version of the manuscript.
